# Musculoskeletal Ultrasound as a Diagnostic Tool for Sports-Related Hip Injuries

**DOI:** 10.7759/cureus.88318

**Published:** 2025-07-19

**Authors:** Zayd Chishti, Laura A Szalacha, Eric E Coris

**Affiliations:** 1 Medicine, University of South Florida Morsani College of Medicine, Tampa, USA; 2 Family Medicine, University of South Florida Morsani College of Medicine, Tampa, USA

**Keywords:** diagnostic musculoskeletal ultrasound, dynamic ultrasound, hip injuries, hip pain in athletes, hip pathology, hip ultrasound, musculoskeletal ultrasound, point-of-care ultrasound (pocus), sports-related hip injuries, sports ultrasound

## Abstract

Musculoskeletal ultrasound (US) is a valuable tool for assessing hip pain due to its wide availability, lack of radiation exposure, and cost-effectiveness. In evaluating sports injuries, dynamic US examination provides substantial benefits in diagnostic clarity and precision. The high prevalence of hip and pelvis injuries among athletes, coupled with the structural complexity and deep position of the hip joint, makes US a practical skill and an indispensable tool in the diagnosis of hip-related injuries. This paper aims to review the normal ultrasonographic anatomy of the hip joint and surrounding structures, outline proper scanning techniques, and delineate common intra-articular and extra-articular pathologies associated with sports participation.

## Introduction and background

As a widely available, radiation-free, and cost-effective tool, musculoskeletal ultrasound (US) serves a valuable role in the assessment of intra-articular and extra-articular causes of hip pain in children and adults [1,2]. In the pediatric population, hip US is widely utilized for screening and diagnosis of developmental dysplasia of the hip, with studies demonstrating increased effectiveness of US screening for developmental dysplasia compared to routine pediatric physical examination [3]. In adults, it is used to delineate between a plethora of hip pathologies and is frequently used to guide therapeutic injections into and around the hip joint.

In the evaluation of sports injuries, dynamic point-of-care US examination and contralateral comparison provide substantial benefits in diagnostic clarity and precision. For extra-articular injuries, dynamic visualization can differentiate between tendinosis and tears, better characterize fluid collections through the use of movement and compression, and elucidate mechanisms behind a snapping hip, all advantages over magnetic resonance imaging (MRI) [4]. In the intra-articular space, joint effusions are easily detected by US. Furthermore, studies have demonstrated that, although not the gold standard, US evaluation can be beneficial for detecting femoroacetabular impingement (FAI), a common source of hip and groin pain affecting both athletes and non-athletes [5].

When utilized to guide procedures, US not only provides an enhanced level of precision, which is directly linked to procedure efficacy and patient safety, but also offers various other benefits, including dynamic visualization, the possibility for repeated scans, contralateral comparison, quantification of soft tissue abnormalities, and non-invasiveness for the patient [6]. Alternative forms of guidance for hip injections, such as landmark guidance, do not offer the same benefits. A systematic review and meta-analysis comparing US-guided injections to landmark-guided injections found that US-guided injections are significantly more accurate [7]. Further, traditional forms of image guidance, like fluoroscopy, once prevalent, have predominantly yielded to the widespread adoption of US in clinical practice. The increased cost, decreased availability, and radiation exposure of fluoroscopic guidance are key factors responsible for this shift [7].

Some estimates suggest that 5% to 6% of sports injuries in adults and 10% to 24% in children originate in the hip and pelvis [4]. These data, combined with the structural complexity and deep position of the hip joint, make proficiency in hip US a practical skill and an indispensable tool in the management of hip-related injuries.

This paper aims to review the normal ultrasonographic anatomy of the hip joint and surrounding structures, outline proper scanning techniques, and delineate common intra-articular and extra-articular pathologies associated with sports participation.

## Review

General technique

When performing a US examination of the hip, it is essential to consider all four anatomical compartments: anterior, medial, lateral, and posterior. Adequate assessment of each region requires specific patient positioning to allow for proper visualization of the relevant structures. Transducer selection should constitute an appropriate balance of image resolution and depth of penetration, as high-frequency transducers provide superior resolution at the expense of image depth, and vice versa. While high-frequency (7.5-20 MHz) linear transducers are generally the most suitable for imaging superficial musculoskeletal structures, such as tendons, ligaments, and small joints, low-frequency probes (3.5-5 MHz) are preferred for imaging deeper joints, such as the hip [8]. However, this may vary widely depending on the patient's body habitus. Higher-frequency probes may be beneficial for examining pediatric patients due to the relatively superficial position of the hip joint, while lower-frequency transducers may be necessary for obese patients to attain sufficient depth of penetration [8]. Additionally, lower-frequency curvilinear probes are preferred for needle tracking during US-guided injections due to their deeper penetration and wider far-field image, as well as enhanced needle visualization resulting from the diverging beams intersecting with the needle shaft at oblique angles [9]. Transducer orientation depends heavily on the structures being examined and the suspected pathologies being assessed for. For instance, a proximal hamstring tendon tear with retraction distally would be visualized most clearly in the long axis. Conversely, the contents of the femoral neurovascular bundle in the femoral triangle are best appreciated in the transverse plane. Imaging structures in both longitudinal and transverse views is crucial to ensure that subtle signs of pathology are not overlooked.

Anterior hip

Sonographic Anatomy

The anterior hip evaluation is typically conducted first, with the patient in a supine position with the hip and knee extended. The location of the hip joint can be approximated by starting at the anterior superior iliac spine (ASIS), which is readily palpable, and moving slightly inferior and medial. Placing the transducer in an oblique longitudinal plane over the femoral neck enables optimal visualization of the anterior joint recess, most clearly seen as a hypoechoic space over the femoral head/neck junction. This space should measure less than 7-8 mm from the femoral bone to the joint capsule in a normal hip without an effusion [6]. Shifting cranially to the femoroacetabular joint, the bony cortices of the acetabular rim and rounded femoral head should be visible. A thin anechoic border along the cortex of the femoral head may also be seen, representing hyaline articular cartilage [6]. The fibrocartilaginous acetabular labrum can be seen as a homogeneously hyperechoic triangular structure originating from the acetabular rim and extending over the femoral head. Only the anterior superior labrum is visible on US. Immediately superficial to the labrum, the iliofemoral ligament of the joint capsule appears as a thin, fibrillar hyperechoic band, which extends from the acetabulum just inferior to the anterior inferior iliac spine (AIIS) to the intertrochanteric line of the femur [10].

More superficially over the femoroacetabular joint, the iliopsoas myotendinous complex can be seen. The iliopsoas muscle appears as a thick hypoechoic structure passing over the anterior surface of the joint, while the conjoint tendon, formed from the fusion of the psoas major and iliacus tendons, appears as a hyperechoic fibrillar structure located in the medial and posterior aspect of the muscle belly. As such, with the probe in a slightly medial position in the long axis, the iliopsoas tendon can be seen as it passes over the acetabular labrum medially [6]. The iliopsoas bursa, which lies between the muscle and the anterior joint capsule, is not typically apparent on US in a non-pathological state. The distal insertion of the iliopsoas tendon onto the lesser trochanter is difficult to observe in the supine position, so the hip must be externally rotated slightly and the probe placed over the medial aspect of the femur over the lesser trochanter. The lesser trochanter can be identified while shifting distally due to the hyperechoic appearance of the bone cortex with the insertion of the hyperechoic fibers of the iliopsoas tendon. This portion of the assessment can also be performed during the medial compartment evaluation.

In the transverse oblique view over the femoral head and neck, the bony cortices of the femoral head and acetabular rim should again be identified first. Directly superficial to the femoral head and its associated anechoic layer of hyaline cartilage, the hyperechoic band representing the joint capsule should be present. Moving slightly proximal to the hip joint, the iliopsoas muscle should appear as a speckled hypoechoic structure with the hyperechoic iliopsoas tendon located in a medial and posterior position. At some levels, the individual components of the iliopsoas muscle may be discernible, including the medial and lateral iliacus fibers and the psoas major, separated by echogenic fascial planes. The iliopsoas tendon notably passes over the iliopectineal eminence, at the junction of the ilium and pubis bones [10].

The other muscles of the anterior hip can be examined by placing the transducer directly over the ASIS. The hyperechoic ASIS appears in the immediate near field with a deep acoustic shadow in the far field due to its very superficial position. In the transverse view, the short tendons of the tensor fascia latae (TFL) laterally and the sartorius medially can be seen attaching to the ASIS. In some cases, the lateral femoral cutaneous nerve may also be seen at this position, appearing as a small circular structure immediately medial to the ASIS and superficial to the sartorius tendon, though the anatomical course of this nerve is highly variable [11]. Compression of this nerve is responsible for meralgia paresthetica, a condition characterized by neuropathic symptoms, including hypoesthesia, hyperesthesia, and dysesthesia, affecting the anterolateral thigh. Further medial and deep to the sartorius, the iliopsoas muscle is seen at this level. Rotating to the sagittal plane over the ASIS and moving inferiorly in a more lateral position, the fibers of the TFL are seen originating from the ASIS and proceeding distally to pass over the greater trochanter and ultimately insert into the anterior border of the fascia lata, just superficial to the vastus lateralis. In the lateral position over the ASIS, the muscle fibers of the gluteus medius and gluteus minimus may also be seen deep to the TFL as they course towards their insertion at the greater trochanter.

The femoral neurovascular bundle, located within the femoral triangle, consists of the femoral nerve, common femoral artery, and common femoral vein, arranged from lateral to medial. These structures are best appreciated in the transverse oblique plane medial to the femoroacetabular joint. The femoral vein is typically the largest of the three structures and can be distinguished from the femoral artery during examination by its compressibility. The pulsatile femoral artery is positioned directly lateral to the vein. Lateral to the femoral artery, the femoral nerve can be identified by its distinctive honeycomb appearance, arising from the presence of multiple hypoechoic fascicles interspersed with echogenic connective tissue. Immediately deep to the neurovascular bundle, the iliacus muscle is situated laterally, while the pectineus muscle lies medially. The hyperechoic cortex of the pubis bone may also be seen deep to the pectineus muscle.

The rectus femoris tendon and myotendinous junction can be evaluated by identifying the AIIS. This can be achieved by tracking the acetabular rim laterally until the hyperechoic bone of the AIIS with its deep acoustic shadow is visualized, along with an attached fibrillar hyperechoic tendon. The rectus femoris has two distinct tendons at its origin: a direct (straight) head originating at the AIIS and an indirect (reflected) head originating from the superolateral acetabular rim. The tendons form a conjoint tendon a few centimeters distal to the AIIS origin [10]. In its longitudinal axis, the hyperechoic fibrillar direct tendon arises from the AIIS and continues inferiorly. The myotendinous junction can be appreciated distally. During this evaluation, the indirect tendon produces an acoustic shadow where it intersects the direct tendon at an oblique angle, which can be mistaken for pathology. To adequately visualize the fibers of the indirect tendon, the transducer must be adjusted to a lateral-to-medial oblique position in the transverse plane due to its path emerging from the acetabular rim [10]. The short-axis assessment of the rectus femoris requires locating the AIIS landmark again. The hyperechoic direct tendon is seen at its origin. Translating the probe caudally, the myotendinous junction is encountered, with the muscle fibers arising from the lateral aspect of the tendon. The muscle belly of the rectus femoris begins to progressively enlarge distally between the muscle bellies of the TFL laterally, the sartorius superficially and medially, and the iliopsoas deep and medially, all of which can be distinguished due to their surrounding hyperechoic fascial planes. Shifting further caudally to the proximal thigh, the central aponeurosis of the rectus femoris, which runs longitudinally through the muscle belly, can be visualized. This is the continuity of the indirect tendon, while the superficial aponeurosis is the extension of the direct tendon [12]. It appears as a hyperechoic linear structure centrally located within the muscle belly. The three vastus muscle bellies, together with the rectus femoris, which comprise the quadriceps, can also be visualized at this level, again distinguishable by their hyperechoic surrounding fascia.

Joint Effusion

Due to the deep position of the hip joint, the presence of an effusion cannot be reliably diagnosed via a standard clinical examination, though concomitant clinical signs may be present in some cases. Thus, US imaging serves a vital role in detecting hip joint effusions. The presence of an intra-articular effusion can be indicative of various underlying pathologies, including trauma, inflammatory or septic arthritis, osteoarthritis, and avascular necrosis, some of which may present with systemic signs or functional limitations, including difficulty or pain with weight-bearing [10,13]. Among athletes, acetabular labral tears or traumatic osteochondral lesions after hip dislocation or subluxation can result in joint effusion [14,15]. On US, a joint effusion appears as a hypoechoic or anechoic pocket of fluid distending the anterior capsule. This is best observed in the oblique longitudinal plane over the femoral head/neck junction. The echogenicity of the fluid varies depending on its composition, which differs, for example, following traumatic versus infectious etiologies [16]. A simple joint effusion typically appears homogenously anechoic, whereas other complex effusions (e.g., infection, trauma, hemorrhage) may display varying echogenicity [13]. Quantitatively, an effusion has been defined as a ≥7 mm distance between the femoral neck and anterior joint capsule or a ≥1 mm distension compared to the contralateral side [17]. A significant complicating factor, however, is the possibility for synovitis to be mistaken for a fluid collection, as a thickened synovium can also distend the anterior capsule [16]. One method to differentiate synovitis from a true joint effusion capitalizes on the association between synovitis and local hypervascularity [16]. Power Doppler US can illuminate hyperemia in the synovium, which would be more indicative of synovitis [13]. Another potential pitfall is improper patient positioning during the examination, as an internally rotated hip can distort the capsule, mimicking a distended anterior recess [10]. Ultimately, despite the utility of US in the setting of a suspected joint effusion, it must not be used to clinically rule out an infected joint, as the absence of anterior distension on US is not a reliable indicator when compared to an arthrocentesis, the traditional gold standard [18]. An anechoic effusion on US should also be further investigated with diagnostic aspiration as a potentially septic effusion [13]. Furthermore, anterior distension on US is unreliable in confirming the presence of an effusion, likely due to the previously discussed imaging complication introduced by synovitis [16,18].

US-guided hip joint aspiration and injection may be performed in the setting of a joint effusion in athletes. The transducer is placed over the joint in an oblique longitudinal plane by first identifying the femoral neurovascular bundle and then translating laterally. The point of entry is typically lateral to the femoral artery and 2 cm inferior to the inguinal ligament [19]. Intra-articular medications, commonly local analgesics or corticosteroids, may be delivered for diagnostic or therapeutic purposes.

Labral Tears

The acetabular labrum serves as a suction seal for the hip joint, maintaining a negative intra-articular pressure to resist distractive forces and ensuring uniform distribution of synovial fluid within the joint [20]. It also increases the articular surface area, resulting in decreased forces applied to the chondral surface [20]. Studies have found that 22% of athletes with groin pain and 55% of patients with hip pain of unknown cause were found to have labral tears [21,22]. Asymptomatic labral tears are also common in athletes, with one youth sports study reporting tears in up to 89% of asymptomatic participants aged 16 and older and up to 56% of those younger than 16 years [23]. On US, only the anterior aspect of the labrum is clearly visualized, and existing data suggest that the anterior and anterosuperior regions of the labrum are most commonly affected [24]. In athletes, sudden twisting or pivoting movements place the labrum at an increased risk for tearing. Specifically, a combined hyperextension and external rotation mechanism is associated with tears of the anterior labrum [24]. It may also be the case that any extremes of hip ranges of motion recruit the weight-bearing role of the labrum, making it more prone to tearing [25]. FAI is also well-described in the literature as a risk factor for labral tears [26]. A notable association exists between the development of FAI and cam morphology, as well as participation in power sports such as basketball and gymnastics, during adolescence prior to puberty [27]. This phenomenon may be attributable to increased forces exerted on the open growth plate, resulting in reactive bone overgrowth at the head/neck junction [27]. Clinically, FAI is often elicited on physical examination by reproducing hip pain through combined flexion, adduction, and internal rotation of the hip joint [26].

On US, hypoechoic clefts in the labrum or detachment from the acetabular rim are indicative of tearing. Associated paralabral cysts may be seen near attachment points of the labrum to the acetabular rim, appearing hypoechoic with smooth, rounded borders [10]. Dynamic examination may be required to appreciate clefts within the labrum or paralabral cysts.

In contrast to US, MRI provides complete visualization of the acetabular labrum, making it the preferred imaging modality for the diagnosis of labral tears. However, one study found that, despite exhibiting a lower sensitivity for detecting anterosuperior labral tears compared to MRI, US imaging demonstrated a higher specificity and PPV [28]. Despite the limitation of visualizing only the anterosuperior labrum, this suggests that US may offer some clinical value in these specific cases, particularly due to its relative efficiency and cost-effectiveness.

Iliopsoas Tendinopathy, Bursitis, and Internal Snapping

Iliopsoas-related pathology has been reported as the primary cause of chronic groin pain in 12% to 36% of athletes based on physical examination findings [29-31]. One large retrospective study found changes in MRI signal intensity in the iliopsoas in 21% of athletes with groin pain [32]. Partial tendon tears and muscle strains tend to be the most common pathology in younger individuals, particularly due to sports-related injuries, whereas complete avulsions with detachment of the lesser trochanter are occasionally seen in the pediatric population and the elderly [33]. Athletes participating in sports involving jumping and kicking may be more affected [34]. In tendinopathy, the iliopsoas tendon appears swollen and hypoechoic with a loss of fibrillar pattern [13].

Extra-articular snapping of the iliopsoas tendon, or internal snapping, typically affects young adults, especially those involved in sports that require repetitive hip abduction, such as ballet dancing [35]. Internal snapping may be a consequence of intra-articular pathology, such as loose bodies or labral tears, or extra-articular injuries, often those involving the iliopsoas myotendinous unit [36]. Dynamic US examination can elucidate the mechanism behind internal snapping. In the transverse plane, with the hip in the neutral position, the rounded, hyperechoic iliopsoas tendon is seen within the posteromedial aspect of the hypoechoic muscle overlying the superior pubic ramus. When the affected hip is flexed, abducted, and externally rotated, the tendon slides laterally and anteriorly over the muscle, causing some muscle fibers to become trapped between the tendon and the superior pubic ramus [37]. Subsequently, during the return to the neutral position, the tendon follows a reverse path, medially and posteriorly, until the muscle fibers are released, at which point it snaps abruptly against the superior pubic ramus [37].

The iliopsoas bursa is the largest bursa in the body, located between the iliopsoas tendon and the anterior joint capsule. It lies between the femoral vessels medially and the iliopsoas muscle laterally in the transverse plane [6]. Iliopsoas bursitis may present as a consequence of intra-articular injury or iliopsoas-related pathology, including tendinopathy and internal snapping. In the transverse plane on US, the distended bursa appears as a well-defined hypoechoic or anechoic space.

Tensor Fascia Latae Tendinopathy

TFL tendinopathy is a common overuse injury in athletes [38]. Due to the joint role of the TFL and the iliotibial band (ITB) in stabilizing the flexed or partially flexed knee during walking or running, tendinopathy of the TFL typically affects runners [38]. This injury may be associated with tenderness over the ASIS on examination [16]. On US, the affected tendon appears enlarged and hypoechoic, likely a consequence of repetitive microtrauma with overuse [38].

Rectus Femoris Tendinopathy

The rectus femoris, which extends across both the hip joint and the knee joint, is the most frequently injured muscle of the anterior thigh [13]. The mechanism of injury typically involves a forceful eccentric contraction of the muscle or an abrupt acceleration or deceleration, often seen in kicking sports, such as soccer or rugby, or in sprinters [39]. During a kicking motion, the proximal tendon is fully stretched when the hip is extended and the knee is flexed, placing it at an increased risk for injury during the subsequent rapid contraction [39]. Furthermore, any abrupt halt in the kicking motion, particularly when met with an opposing force, significantly increases the risk of injury [39]. Eccentric forces applied to the proximal rectus femoris during sprinting are also associated with avulsions [39].

Skeletally immature athletes may suffer apophyseal injuries of the proximal rectus femoris at the AIIS [13]. US can demonstrate a tendon avulsion and a displaced bony fragment, which may be associated with anechoic fluid [10]. In mature athletes, proximal tendon injuries are less common [34]. The distal myotendinous junction and the myotendinous junction of the indirect head, known as the central aponeurosis, are frequent sites of injury in adults [13]. Injuries at the myotendinous junction can range from partial tears, with the tendon appearing thickened and hypoechoic with a loss of fibrillar pattern, to complete tears with varying levels of retraction [34]. The latter case would demonstrate an anechoic gap in the tendon fibers with an associated hematoma on US [16]. An injured central aponeurosis may be poorly discernible on US but is associated with thickening, variable echogenicity, and surrounding edema [34]. Calcific tendinopathy of the direct head of the rectus femoris, often associated with repetitive trauma, can also be appreciated on US as an irregular hyperechoic structure within the otherwise smooth, fibrillar pattern of the tendon [13,34].

Medial hip

Sonographic Anatomy

Examination of the medial compartment is conducted with the patient in a frog-leg position, with the hip abducted and externally rotated and the knee flexed [40]. The muscles of this compartment include the gracilis, adductor longus, adductor brevis, and adductor magnus, from superficial to deep. With the probe in a transverse orientation over the bulk of the adductor musculature, the muscle layers can be visualized in their short-axis views, separated by fascia. The superficial layer consists of the adductor longus laterally and the gracilis medially. The adductor longus appears rounded or oval-shaped, while the gracilis exhibits a slender appearance. The adductor brevis comprises the intermediate layer and appears thick and broad. The deep layer is the broad adductor magnus muscle belly. Within the fascial layers separating the adductor muscles lie branches of the obturator nerve, which may be discernible as small, dark tubular structures in the hyperechoic fascia, depending on the quality of the US image. Specifically, the anterior branch of the obturator nerve lies in the fascia between the adductor brevis muscle and adductor longus or pectineus muscles, while the posterior branch is located between the adductor brevis and adductor magnus muscles. Rotating to the long-axis view over the adductors, the three layers are seen again, from superficial to deep: adductor longus, adductor brevis, and adductor magnus. The gracilis may be seen in a more medial position and can be mistaken for the adductor longus due to their similar appearances in the long axis. To prevent misidentification, it is recommended to first identify these muscles in their short-axis views before transitioning to the longitudinal view. Scanning superomedially toward the pubis, which is identified by its hyperechoic cortex and deep acoustic shadow, the origins of the adductor tendons can be observed. At its origin at the body of the pubis, just lateral to the pubic symphysis and inferior to the pubic crest, the adductor longus tendon appears as a triangular hypoechoic structure [10,12].

Adductor Tendinopathy

Hip adductor injuries are prevalent in sports requiring kicking or sudden changes in direction [41]. Adductor injuries are estimated to account for approximately 22% of hip injuries in National Basketball Association players, while in professional soccer players, 23% of all muscle injuries were found to be adductor-related [42,43]. The mechanism of adductor injury typically involves a combined hip abduction with abdominal wall hyperextension, sometimes accompanied by forced external rotation of the leg [44]. The adductor longus and gracilis are most frequently affected [45].

In adductor tendinopathy, the tendon appears hypoechoic and thickened on US. In partial tears, the tendon origin at the pubis appears heterogeneous and poorly defined with a discontinuity in its fibers, while a complete rupture demonstrates separation from the pubis with possible retraction [16]. Acute injuries may be associated with a hematoma near the pubis, contributing to the heterogeneous appearance [16]. Cortical irregularities of the pubis or calcification may also be detected on US [13].

Lateral hip

Sonographic Anatomy

To examine the lateral hip, the patient lies in a lateral decubitus position with the hip slightly flexed [10]. This position optimizes visualization of the hip abductors, ITB, associated bursae, and greater trochanter. The echogenic gluteus medius and minimus tendons can be assessed at their insertion points on the greater trochanter, the key bony landmark for the lateral hip examination, which appears as a hyperechoic line. Positioning the transducer in a transverse plane over the greater trochanter, perpendicular to the femoral shaft, allows for clear visualization of the abductor tendon insertions onto the distinct trochanteric facets. The gluteus minimus tendon inserts onto the anterior facet of the greater trochanter, appearing hyperechoic and fibrillar. The gluteus medius is seen as a curvilinear fibrillar band over the lateral and superoposterior facets of the greater trochanter. Distinguishing the anterior facet from the lateral and superoposterior facets on US requires first identifying the apex of the greater trochanter, which is seen as a raised peak in the bony cortex [10]. This landmark serves as a border, separating the anterior facet anteriorly from the lateral and superoposterior facets posteriorly. Over the superoposterior facet of the greater trochanter, superficial to the gluteus medius tendon, the hypoechoic anterior aspect of the gluteus maximus is observed as it tapers anteriorly toward its insertion into the ITB fibers, which appear as a thin hyperechoic stripe [10]. The ITB fibers are joined by the anterior fibers of the gluteus maximus and posterior fibers of the TFL as they proceed caudally, superficial to the greater trochanter, gluteus medius, and gluteus minimus tendons, toward their insertion onto the tibia [10]. Several bursae are present between the tendinous layers over the greater trochanter. The greater trochanteric bursa is located between the gluteus medius tendon and the overlying ITB. The subgluteus medius bursa lies deep to the gluteus medius tendon, while the subgluteus minimus bursa is positioned deep to the gluteus minimus tendon. Though these potential spaces are generally not visualized on US, they may appear as thin hypoechoic pockets when distended. In the longitudinal plane, the gluteus minimus muscle is seen as the deepest layer, with its fibers originating from the posterior ilium deep to the gluteus medius and inserting onto the anterior facet of the greater trochanter. The gluteus medius muscle fibers are seen just superficially in this position. To adequately assess the insertion of both tendons onto their individual facets in longitudinal section, the transducer should be shifted over the greater trochanter between superoposterior and anteroinferior positions [13]. Identifying the tendons in the transverse view before rotating to the longitudinal view may aid in distinguishing between the two at their insertions. In addition to the actual positioning of the probe, angling it with an anterior-to-posterior tilt while evaluating the gluteus minimus tendon and a posterior-to-anterior tilt while imaging the gluteus medius tendon may help reduce anisotropic artifact in the long-axis views. The hyperechoic linear ITB overlies the greater trochanter and abductor tendons.

Greater Trochanteric Pain Syndrome

Though previously thought to be primarily a consequence of trochanteric bursitis, greater trochanteric pain syndrome (GTPS) as a clinical diagnosis now encompasses various pathologies of the lateral peritrochanteric region of the hip, including gluteus medius and minimus tendinopathy, trochanteric bursitis, and external snapping of the ITB [46]. Recent data suggest that pathologies of the gluteal tendons and ITB are more frequently implicated as primary etiologies of GTPS rather than isolated trochanteric bursitis [47]. The condition classically presents as chronic pain in the peritrochanteric region, often worsened with hip abduction, and tenderness to palpation over the greater trochanter [34]. GTPS is reported to affect between 10% and 25% of the general population, with a greater prevalence in women than men [46]. Middle-aged and elderly women are most frequently affected [16]. Risk factors such as a history of low back pain or leg length discrepancy have been associated with an increased prevalence of GTPS [48]. Among athletes, runners who adduct beyond midline or run on uneven surfaces are believed to be at a greater risk for developing trochanteric bursitis [49].

The pathology of the gluteal tendons at their insertion onto the greater trochanter is appreciable on US. This can range from tendinopathy to partial or complete tears. A heterogeneous tendon with hypoechoic thickening is characteristic of gluteal tendinopathy on US. Irregular hyperechoic foci at the insertion site may represent calcification. Partial-thickness tears most frequently involve the deep and anterior fibers of the gluteus medius and appear as a focal discontinuity in the fibrillar pattern or anechoic clefts [50]. Complete tears demonstrate a retracted tendon with a bare trochanteric facet and anechoic fluid occupying the intervening space [40]. The "bald" greater trochanter appearance signifying the absence of the tendon from its corresponding facet may be appreciated in both transverse and longitudinal views [51]. It is postulated that partial tearing of the anterior fibers can lead to further tearing, which, extending posteriorly, can ultimately result in a complete avulsion [50]. Gluteus minimus tears rarely occur in isolation and frequently are associated with concomitant gluteus medius tears [50]. Cortical irregularities of the greater trochanter can be visualized in many cases [50].

Though rarely present in isolation, trochanteric bursitis is occasionally detected on US as a secondary manifestation [47]. It appears as a hypoechoic collection deep to the gluteus maximus fibers and ITB and superficial to the superoposterior facet of the greater trochanter and the gluteus medius tendon insertion at the lateral facet [34].

External Snapping

External snapping of the hip refers to a transient subluxation of the anterior fibers of the gluteus maximus and the ITB over the greater trochanter [35]. Though most cases are idiopathic, younger individuals, particularly athletes in their teens and twenties, are most commonly affected [36]. Dancers and other athletes frequently performing movements involving extreme ranges of motion are particularly susceptible and also exhibit a greater prevalence of painful snapping [52].

Dynamic US is useful in demonstrating the abnormal movement of structures producing the snapping. With the patient in a lateral decubitus position, the lateral hip structures of the symptomatic side are examined in longitudinal and transverse views. In cases of painful external snapping, the ITB may appear thickened and hypoechoic, and trochanteric bursitis may also be present [35]. During normal flexion and extension of the hip, the anterior fibers of the gluteus maximus and the ITB pass smoothly over the lateral aspect of the greater trochanter [35]. When external snapping is present, a transverse US view over the greater trochanter demonstrates the gluteus maximus fibers and ITB temporarily impeded by the posterolateral aspect of the greater trochanter during the early phase of flexion [35]. During late flexion, these fibers are abruptly released anteriorly over the lateral aspect of the greater trochanter, producing a snap [35]. Snapping may also occur during the subsequent extension phase when the fibers slide posteriorly over the greater trochanter [35]. External snapping may also be elicited with the patient standing and leaning on the affected side during the extension phase, as this more tightly secures the ITB fibers over the posterolateral greater trochanter [35].

Posterior hip

Sonographic Anatomy

The gluteus maximus muscle fibers, proximal hamstrings, and sciatic nerve can be evaluated via the posterior approach with the patient in a prone position with hips and knees extended. The ischial tuberosity is the primary bony landmark of the posterior compartment. With the transducer in a transverse plane just cranial to the ischial tuberosity, the thick fibers of the gluteus maximus muscle are readily visible in their long-axis orientation. The ordinarily invisible ischiogluteal bursa is present between the gluteus maximus and the ischial tuberosity. Adipose tissue is present in the near field, and the ischial tuberosity is seen deep, its cortex appearing as a hyperechoic curvilinear structure. When examining patients with greater adiposity in this region, a lower-frequency probe may be necessary to visualize these structures properly. Shifting the probe caudally toward the gluteal crease, the origins of the hamstring tendons can be appreciated. The conjoint tendon of the semitendinosus and biceps femoris long head originates from the inferomedial aspect of the ischial tuberosity [53]. The thicker semimembranosus tendon arises from the superolateral aspect of the ischial tuberosity, coursing anterior and medial to the conjoint tendon [53]. At the level of the ischial tuberosity, the conjoint tendon and semimembranosus tendon cannot be clearly distinguished but appear together as an echogenic bundle over the ischial tuberosity. The sciatic nerve may be detectable lateral to the hamstring tendons, posterior to the gluteus maximus [6]. Translating the probe caudally, the conjoint tendon of the semitendinosus and biceps femoris long head can be distinguished from the semimembranosus tendon. The conjoint tendon has a more superficial and lateral position and appears as a hyperechoic structure separating the hypoechoic muscle bellies of the biceps femoris laterally and semitendinosus medially. The semimembranosus has a large echogenic aponeurosis attached to the medial side of its "tadpole-shaped" tendon, from which the muscle belly arises medially [12]. At this level, the fascicular honeycomb appearance of the sciatic nerve is more readily apparent just deep to the long head of the biceps femoris. Deep to all of these structures, the hamstring portion of the adductor magnus muscle is seen. In this view, the hyperechoic fascial planes of the hamstring muscles and the adductor magnus are oriented to create a distinctive landmark, often referred to as the "Mercedes-Benz sign" [13]. At the distal thigh, just above the popliteal fossa, the semitendinosus and semimembranosus are positioned medially and can be distinguished in their transverse view using another sonographic landmark known as the "cherry on the cake sign" [13]. They are described as such due to the superficial position of the thin semitendinosus tendon lying over the broad semimembranosus muscle belly. In the longitudinal plane at the level of the gluteal crease, the hyperechoic ischial tuberosity is located, and the hyperechoic fibrillar appearance of the hamstring tendon origins is appreciated. Again, at this level, the conjoint tendon and semimembranosus tendon cannot be clearly separated.

The piriformis muscle can also be visualized with the posterior approach. Its fibers run nearly parallel to those of the gluteus maximus. The sonographic technique to locate the piriformis begins with placing the transducer over the palpable posterior superior iliac spine (PSIS). The transducer is then translated laterally over the iliac wing, bringing into view the continuous hyperechoic bone deep to the gluteus medius and minimus muscles. The transducer is then shifted caudally until the hyperechoic ilium disappears, suggesting the appearance of the greater sciatic notch, which the piriformis traverses [40]. The fibers of the piriformis are visible at this level, running cephalomedial to caudolateral, deep to the fibers of the gluteus maximus [40]. The triangular shape, oblique orientation, and unique echogenic profile, compared to the gluteus maximus, enable the identification of the piriformis. The transducer is then obliqued at this level by rotating laterally to align with the piriformis fibers. Passive internal and external rotation of the hip can confirm the presence of the piriformis fibers gliding below the gluteus maximus. The inferior gluteal artery and sciatic nerve may be identified beneath the piriformis.

Hamstring Tendinopathy and Strains

Hamstring injuries are some of the most common lower extremity injuries in athletes, ranging from proximal tendinopathy to acute muscle strains and tears [54]. Acute hamstring strains can be broadly categorized into two groups based on the mechanisms of injury. Type I strains predominantly affect the proximal myotendinous junction of the long head of the biceps femoris [54]. These injuries typically occur as a result of rapid acceleration, deceleration, or a change in direction and can also occur during sprinting, as the hamstrings eccentrically contract during the terminal swing phase of running [54]. This movement occurs routinely in various sports, including football, basketball, soccer, and sprinting [16]. A 10-year study of National Football League training camp injuries found that hamstring strains were the second most prevalent injury behind knee sprains [55]. Type II injuries occur as a consequence of excessive lengthening of the hamstrings, typically with extreme hip flexion and knee extension [54]. This mechanism of injury is frequently observed in skiers, water skiers, ballet dancers, gymnasts, and skaters [56,57]. These injuries most commonly affect the proximal semimembranosus tendon, close to the ischial tuberosity [57]. Like the rectus femoris, the hamstrings are particularly susceptible to rapid muscle lengthening and, thus, strains because they span both the hip and knee joints.

On US, musculotendinous strains demonstrate a loss of fibrillar pattern, anechoic clefts, and surrounding edema [34]. Though the presence of edema or hemorrhage can aid in identifying moderate to severe acute musculotendinous strains, milder strains may be more difficult to visualize, especially in patients with larger body habitus [34].

Proximal hamstring tendinopathy, like other tendinopathies, is associated with hypoechoic thickening. Calcific tendinopathy may be detected as irregular hyperechoic structures at the tendon origin. In skeletally immature athletes, displaced avulsion fragments may be seen on US in proximal tendon injuries. A partial tendon tear exhibits irregularities or minor discontinuities in the fibrillar pattern and anechoic clefts [10]. Complete tears present with a gap occupied by fluid or hematoma. The long-axis view is ideal for visualizing the anechoic gap and retraction of the tendon fibers from the origin of the hyperechoic ischial tuberosity. The lack of a tendon bundle above the ischial tuberosity in the short-axis view is indicative of a complete tear with retraction [58]. Ischiogluteal bursitis may co-occur with proximal hamstring pathology, with the distended bursa appearing as a hypoechoic or anechoic fluid collection superficial to the ischial tuberosity.

Piriformis Syndrome

Piriformis syndrome has been well established as a common cause of extraspinal sciatica [59]. It is understood to result from overuse or repetitive trauma, and the resultant symptoms are a consequence of compression and irritation of the sciatic nerve [59]. These symptoms typically include posterior hip and buttock pain, along with sensory symptoms in the sciatic nerve distribution, including numbness and paresthesias [59]. Given that a substantial proportion of lower back pain is of unexplained etiology, considering piriformis syndrome in the evaluation of nonspecific lower back pain in athletes may be warranted [60].

Piriformis syndrome is characterized on US by a hypoechoic, swollen appearance of the muscle fibers [40]. This finding is corroborated by comparing the affected side to the asymptomatic contralateral side.

## Conclusions

The comprehensive evaluation of the hip joint using US provides critical insights into various pathologies, particularly in athletes. The ability to dynamically assess and visualize structures in multiple planes makes US an essential tool for achieving diagnostic clarity and precision. With a robust understanding of sonographic hip anatomy, scanning techniques, and common injury patterns, clinicians can improve clinical assessment and optimize patient care.
